# (1*E*,2*E*)-1,2-Bis(2,3,4-trimeth­oxy­benzyl­idene)hydrazine

**DOI:** 10.1107/S160053681003518X

**Published:** 2010-09-15

**Authors:** Malai Haniti S. A. Hamid, Mohammad Akbar Ali, Aminul Huq Mirza, Gan Ai Len, Ray J. Butcher

**Affiliations:** a Faculty of Science, Universiti Brunei Darussalam, Jln Tungku Link, BE 1410, Negara Brunei Darussalam; b Department of Chemistry, Howard University, 525 College Street NW, Washington, DC 20059, USA

## Abstract

The title compound, C_20_H_24_N_2_O_6_, was obtained as an unexpected product by the reaction of hydrazinium dithio­carbazate with 2,3,4-trimeth­oxy­benzaldehyde in refluxing ethanol. The mol­ecule lies on a center of inversion. The crystal packing is stabilized by weak inter­molecular C—H⋯O inter­actions.

## Related literature

The surprising formation of the title hydrazone was probably due to the decomposition of hydrazinium dithio­carbazate in solution resulting in the formation of hydrazine, which then reacted with 2,3,4-trimeth­oxy­benzaldehdye. Hydrazinium dithio­carbaza­tes are known to decompose on heating (Rudorf, 2007 ▶). For the biological activity of Schiff bases, see: Akbar Ali *et al.* (2008 ▶); Chan *et al.* (2008 ▶). For a previous report of the title compound (the X-ray structure was not provided), see: Praefcke *et al.* (1991 ▶). For comparison bond lengths in an aroyl hydrazone, see: Ji *et al.* (2010 ▶).
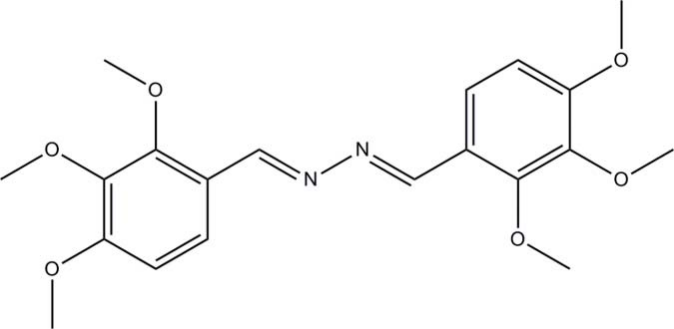


## Experimental

### 

#### Crystal data

C_20_H_24_N_2_O_6_
*M* *_r_* = 388.41
Monoclinic, 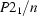

*a* = 10.0380 (9) Å
*b* = 7.0713 (7) Å
*c* = 13.9586 (14) Å
β = 102.800 (2)°
*V* = 966.18 (16) Å^3^
*Z* = 2
Mo *K*α radiation
μ = 0.10 mm^−1^
*T* = 100 K
0.60 × 0.36 × 0.04 mm

#### Data collection

Bruker SMART CCD area-detector diffractometer
Absorption correction: multi-scan (*SADABS*; Sheldrick, 1996 ▶) *T*_min_ = 0.943, *T*_max_ = 0.996
6576 measured reflections
2203 independent reflections
1846 reflections with *I* > 2σ(*I*)
*R*_int_ = 0.033

#### Refinement

*R*[*F* ^2^ > 2σ(*F* ^2^)] = 0.048
*wR*(*F* ^2^) = 0.123
*S* = 1.05
2196 reflections
130 parameters
H-atom parameters constrained
Δρ_max_ = 0.35 e Å^−3^
Δρ_min_ = −0.20 e Å^−3^

### 

Data collection: *SMART* (Bruker, 1998 ▶); cell refinement: *SAINT-Plus* (Bruker, 1998 ▶); data reduction: *SAINT-Plus*; program(s) used to solve structure: *SHELXS97* (Sheldrick, 2008 ▶); program(s) used to refine structure: *SHELXL97* (Sheldrick, 2008 ▶); molecular graphics: *SHELXTL* (Sheldrick, 2008 ▶); software used to prepare material for publication: *SHELXTL*.

## Supplementary Material

Crystal structure: contains datablocks I, global. DOI: 10.1107/S160053681003518X/bt5339sup1.cif

Structure factors: contains datablocks I. DOI: 10.1107/S160053681003518X/bt5339Isup2.hkl

Additional supplementary materials:  crystallographic information; 3D view; checkCIF report

## Figures and Tables

**Table 1:** Hydrogen-bond geometry (Å, °)

*D*—H⋯*A*	*D*—H	H⋯*A*	*D*⋯*A*	*D*—H⋯*A*
C7—H7*B*⋯O3^ii^	0.98	2.54	3.3620 (18)	142
C8—H8*B*⋯O1^iii^	0.98	2.62	3.3735 (19)	134
C8—H8*B*⋯O2^iii^	0.98	2.62	3.5711 (18)	164

Symmetry codes: (ii) 
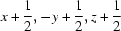
; (iii) 
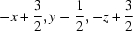
.

**Fig. 1.**
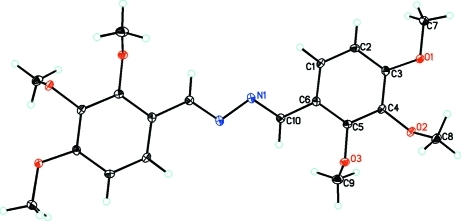
The title compound, C20H24N2O6 with atom labeling. Displacement ellipsoids are at the 50 % probability level.

**Fig. 2.**
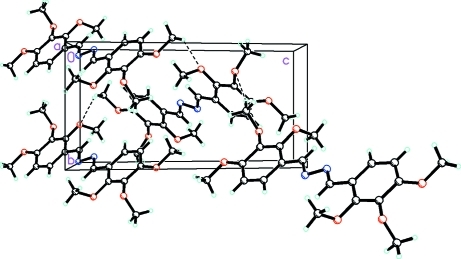
The molecular packing for C20H24N2O6 viewed down the a axis showing the intermolecular C—H···O interactions.

## supplementary crystallographic information

### Comment

The compound, C_20_H_24_N_2_O_6_ (I) was obtained by the reaction of hydrazinium dithiocarbazate and 2,3,4-trimethoxybenzaldehyde in boiling ethanol. The surprising formation of the hydrazone was probably due to the decomposition of hydrazinium dithiocarbazate in solution resulting in the formation of hydrazine, which then reacted with 2,3,4-trimethoxybenzaldehdye to form the corresponding hydrazone (I). Hydrazinium dithiocarbazates are known to decompose on heating (Rudorf, 2007).

Schiff bases have attracted considerable attention because they can act as chelating agents for metal ions and many of them also exhibit useful biological activities (Akbar Ali *et al.*, 2008; Chan *et al.*, 2008). Although the compound has previously been reported its X-ray structure has not been provided (Praefcke *et al.*, 1991). Hydrazones derived from the reactions of hydrazines with aldehydes or ketones are common but bis-hydrazones are not.

The molecular structure of (I) is shown in Figure 1 and its selected bond lengths and angles are given in Table 1. Like most thiosemicarbazones and Schiff bases, the imine moiety in [I] shows an E configuration about the C10—N1 [1.283 (2) Å] and N1A—C10A bonds. The C10—N1 and N1A—C10A bond distances also compare well with C=N double bonds in other related compounds. A comparison of the N(1)—N(1 A) distance [1.413 (3) Å] with that in an aroyl hydrazone [1.377 (3) Å] (Ji *et al.* 2010) shows that the bond is shorter than a single N—N bond (1.44 Å) indicating that a significant π-charge delocalization occurs along the C—N—N—C moiety. As the bond angles C6—C10—N1 (121.68°) and C6A—C10A—N1A (121.68°) are close to that of a *sp*^2^-hybridized carbon atom (*ca* 120°), the molecule does not have a distorted geometry. Due the fact that the molecule lies on a center of inversion the dihedral angle between the two phenyl rings is 0.0°.

Figure 2 shows the packing of (I) in the unit cell. The packing diagram shows that there are intermolecular hydrogen bonds between one of the CH3 hydrogen atoms of one molecule with an ether oxygen of another molecule.

### Experimental

2,3,4-trimethoxybenzaldehyde (0.24 g, 1.24 mmol) dissolved in absolute ethanol (5 ml) was mixed with a solution of hydrazinium dithiocarbazate (0.93 g, 0.66 mmol) in the same solvent (45 ml). After refluxing for two hours, the resulting clear yellow solution was left to stand at room temperature for five days to afford crystalline yellow plates. The crystals were filtered, washed with cold absolute ethanol and dried *in vacuo*. Yield: 0.152 g (63%); m.p. 192–194 °C; IR (KBr, cm^-1^): 2968, 2937, 2832, 1614, 1590, 1494, 1457, 1431, 1410, 1286, 1229, 1199. 1166, 1090, 1023, 1008, 943, 898, 809, 699, 667, 594, 540, 433; ^1^H NMR (400 MHz, CDCl_3_, 30 °C): δ 8.92 (2*H*, s, CH=N), 7.84 (2*H*, d, ArH), 6.76 (2*H*, d, ArH), 3.96 (6*H*, s, OCH_3_), 3.92 (6*H*, s, OCH_3_), 3.90 (6*H*, s, OCH_3_); Anal. Calcd. for C_20_H_24_N_2_O_6_ (388.42): C 61.85, H 6.23, N 7.21. Found: C 62.17, H 6.17, N 7.47%.

IR spectrum was recorded as a KBr disc with 13 mm KBr discs SPECAC accessory on a Perkin-Elmer 1600 F T IR spectrometer. ^1^H NMR spectrum was run in CDCl_3_ on a Varian 400-NMR spectrometer at Universiti Brunei Darussalam. Elemental analysis for C, H and N was done by the Elemental Analysis Laboratory, Department of Chemistry, National University of Singapore. The X-ray data were collected at the X-ray Diffraction Laboratory, Department of Chemistry, National University of Singapore using a Bruker-AXS Smart Apex CCD single-crystal diffractometer.

### Refinement

H atoms were placed in geometrically idealized positions and constrained to ride on their parent atoms with a C—H distances of 0.95 Å and 0.98 Å, *U*_iso_(H) = 1.2*U*_eq_(C).

### Crystal data

**Table:**

C_20_H_24_N_2_O_6_	*F*(000) = 412
*M**_r_* = 388.41	*D*_x_ = 1.335 Mg m^−^^3^
Monoclinic, *P*2_1_/*n*	Mo *K*α radiation, λ = 0.71073 Å
*a* = 10.0380 (9) Å	Cell parameters from 1770 reflections
*b* = 7.0713 (7) Å	θ = 2.8–27.1°
*c* = 13.9586 (14) Å	µ = 0.10 mm^−^^1^
β = 102.800 (2)°	*T* = 100 K
*V* = 966.18 (16) Å^3^	Plate, yellow
*Z* = 2	0.60 × 0.36 × 0.04 mm

### Data collection

**Table:**

Bruker SMART CCD area-detector diffractometer	2203 independent reflections
Radiation source: fine-focus sealed tube	1846 reflections with *I* > 2σ(*I*)
graphite	*R*_int_ = 0.033
ω scans	θ_max_ = 27.5°, θ_min_ = 2.3°
Absorption correction: multi-scan (*SADABS*; Sheldrick, 1996)	*h* = −13→12
*T*_min_ = 0.943, *T*_max_ = 0.996	*k* = 0→9
6576 measured reflections	*l* = 0→18

### Refinement

**Table:**

Refinement on *F*^2^	Primary atom site location: structure-invariant direct methods
Least-squares matrix: full	Secondary atom site location: difference Fourier map
*R*[*F*^2^ > 2σ(*F*^2^)] = 0.048	Hydrogen site location: inferred from neighbouring sites
*wR*(*F*^2^) = 0.123	H-atom parameters constrained
*S* = 1.05	*w* = 1/[σ^2^(*F*_o_^2^) + (0.0608*P*)^2^ + 0.353*P*] where *P* = (*F*_o_^2^ + 2*F*_c_^2^)/3
2196 reflections	(Δ/σ)_max_ < 0.001
130 parameters	Δρ_max_ = 0.35 e Å^−^^3^
0 restraints	Δρ_min_ = −0.20 e Å^−^^3^

### Special details

**Table:**

Geometry. All e.s.d.'s (except the e.s.d. in the dihedral angle between two l.s. planes) are estimated using the full covariance matrix. The cell e.s.d.'s are taken into account individually in the estimation of e.s.d.'s in distances, angles and torsion angles; correlations between e.s.d.'s in cell parameters are only used when they are defined by crystal symmetry. An approximate (isotropic) treatment of cell e.s.d.'s is used for estimating e.s.d.'s involving l.s. planes.
Refinement. Refinement of *F*^2^ against ALL reflections. The weighted *R*-factor *wR* and goodness of fit *S* are based on *F*^2^, conventional *R*-factors *R* are based on *F*, with *F* set to zero for negative *F*^2^. The threshold expression of *F*^2^ > σ(*F*^2^) is used only for calculating *R*-factors(gt) *etc*. and is not relevant to the choice of reflections for refinement. *R*-factors based on *F*^2^ are statistically about twice as large as those based on *F*, and *R*- factors based on ALL data will be even larger.

### Fractional atomic coordinates and isotropic or equivalent isotropic displacement parameters (Å^2^)

**Table:**

	*x*	*y*	*z*	*U*_iso_*/*U*_eq_	
O1	0.62846 (10)	0.45681 (14)	0.85047 (7)	0.0180 (2)	
O2	0.61624 (9)	0.20990 (14)	0.70188 (7)	0.0165 (2)	
O3	0.38623 (10)	0.18335 (14)	0.55439 (7)	0.0175 (2)	
C10	0.15962 (14)	0.4221 (2)	0.54359 (10)	0.0154 (3)	
H10	0.1582	0.3285	0.4945	0.018*	
C1	0.29226 (14)	0.5791 (2)	0.69604 (11)	0.0172 (3)	
H1	0.2190	0.6649	0.6944	0.021*	
C2	0.40646 (15)	0.5899 (2)	0.77231 (10)	0.0180 (3)	
H2	0.4113	0.6834	0.8218	0.022*	
C3	0.51481 (14)	0.4635 (2)	0.77677 (10)	0.0153 (3)	
C4	0.50841 (13)	0.33003 (19)	0.70148 (10)	0.0146 (3)	
C5	0.39310 (14)	0.32122 (19)	0.62483 (10)	0.0147 (3)	
C6	0.28211 (14)	0.44466 (19)	0.62113 (10)	0.0149 (3)	
C7	0.64403 (15)	0.6012 (2)	0.92428 (11)	0.0202 (3)	
H7A	0.6488	0.7252	0.8938	0.030*	
H7B	0.7282	0.5789	0.9740	0.030*	
H7C	0.5657	0.5983	0.9555	0.030*	
C8	0.60816 (16)	0.0407 (2)	0.75753 (11)	0.0218 (3)	
H8A	0.6081	0.0745	0.8256	0.033*	
H8B	0.6871	−0.0401	0.7563	0.033*	
H8C	0.5238	−0.0274	0.7286	0.033*	
C9	0.44808 (17)	0.2382 (2)	0.47521 (11)	0.0250 (4)	
H9A	0.3970	0.3440	0.4393	0.038*	
H9B	0.4466	0.1309	0.4305	0.038*	
H9C	0.5428	0.2769	0.5017	0.038*	
N1	0.05345 (12)	0.52567 (17)	0.53984 (8)	0.0165 (3)	

### Atomic displacement parameters (Å^2^)

**Table:**

	*U*^11^	*U*^22^	*U*^33^	*U*^12^	*U*^13^	*U*^23^
O1	0.0149 (5)	0.0161 (5)	0.0206 (5)	0.0020 (4)	−0.0011 (4)	−0.0043 (4)
O2	0.0135 (5)	0.0140 (5)	0.0223 (5)	0.0028 (4)	0.0046 (4)	0.0010 (4)
O3	0.0191 (5)	0.0148 (5)	0.0188 (5)	−0.0001 (4)	0.0043 (4)	−0.0027 (4)
C10	0.0161 (6)	0.0143 (7)	0.0160 (6)	0.0000 (5)	0.0043 (5)	0.0016 (5)
C1	0.0152 (7)	0.0160 (7)	0.0206 (7)	0.0040 (5)	0.0045 (5)	0.0010 (5)
C2	0.0199 (7)	0.0159 (7)	0.0183 (7)	0.0008 (6)	0.0043 (6)	−0.0041 (5)
C3	0.0119 (6)	0.0155 (7)	0.0175 (7)	−0.0021 (5)	0.0014 (5)	0.0012 (5)
C4	0.0130 (6)	0.0130 (7)	0.0189 (7)	0.0015 (5)	0.0055 (5)	0.0020 (5)
C5	0.0167 (6)	0.0128 (7)	0.0155 (6)	−0.0011 (5)	0.0055 (5)	0.0002 (5)
C6	0.0136 (6)	0.0146 (7)	0.0164 (6)	−0.0006 (5)	0.0028 (5)	0.0025 (5)
C7	0.0203 (7)	0.0190 (7)	0.0196 (7)	−0.0001 (6)	0.0009 (6)	−0.0035 (6)
C8	0.0277 (8)	0.0176 (8)	0.0212 (7)	0.0069 (6)	0.0082 (6)	0.0040 (6)
C9	0.0320 (8)	0.0244 (8)	0.0206 (7)	0.0016 (7)	0.0098 (6)	−0.0024 (6)
N1	0.0146 (6)	0.0185 (6)	0.0154 (6)	−0.0004 (5)	0.0012 (5)	0.0020 (5)

### Geometric parameters (Å, °)

**Table:**

O1—C3	1.3572 (16)	C3—C4	1.4036 (19)
O1—C7	1.4344 (17)	C4—C5	1.3926 (19)
O2—C4	1.3750 (16)	C5—C6	1.4072 (19)
O2—C8	1.4383 (17)	C7—H7A	0.9800
O3—C5	1.3755 (17)	C7—H7B	0.9800
O3—C9	1.4357 (18)	C7—H7C	0.9800
C10—N1	1.2846 (19)	C8—H8A	0.9800
C10—C6	1.4556 (19)	C8—H8B	0.9800
C10—H10	0.9500	C8—H8C	0.9800
C1—C2	1.383 (2)	C9—H9A	0.9800
C1—C6	1.400 (2)	C9—H9B	0.9800
C1—H1	0.9500	C9—H9C	0.9800
C2—C3	1.398 (2)	N1—N1^i^	1.411 (2)
C2—H2	0.9500		
			
C3—O1—C7	117.32 (11)	C1—C6—C10	122.64 (13)
C4—O2—C8	112.18 (11)	C5—C6—C10	119.40 (13)
C5—O3—C9	113.41 (11)	O1—C7—H7A	109.5
N1—C10—C6	121.62 (13)	O1—C7—H7B	109.5
N1—C10—H10	119.2	H7A—C7—H7B	109.5
C6—C10—H10	119.2	O1—C7—H7C	109.5
C2—C1—C6	121.54 (13)	H7A—C7—H7C	109.5
C2—C1—H1	119.2	H7B—C7—H7C	109.5
C6—C1—H1	119.2	O2—C8—H8A	109.5
C1—C2—C3	120.20 (13)	O2—C8—H8B	109.5
C1—C2—H2	119.9	H8A—C8—H8B	109.5
C3—C2—H2	119.9	O2—C8—H8C	109.5
O1—C3—C2	124.83 (13)	H8A—C8—H8C	109.5
O1—C3—C4	115.81 (12)	H8B—C8—H8C	109.5
C2—C3—C4	119.36 (12)	O3—C9—H9A	109.5
O2—C4—C5	119.67 (12)	O3—C9—H9B	109.5
O2—C4—C3	120.47 (12)	H9A—C9—H9B	109.5
C5—C4—C3	119.85 (12)	O3—C9—H9C	109.5
O3—C5—C4	118.84 (12)	H9A—C9—H9C	109.5
O3—C5—C6	119.97 (12)	H9B—C9—H9C	109.5
C4—C5—C6	121.13 (13)	C10—N1—N1^i^	111.38 (15)
C1—C6—C5	117.88 (13)		
			
C6—C1—C2—C3	−0.8 (2)	O2—C4—C5—O3	4.25 (19)
C7—O1—C3—C2	−6.1 (2)	C3—C4—C5—O3	−176.89 (12)
C7—O1—C3—C4	174.78 (12)	O2—C4—C5—C6	−178.71 (12)
C1—C2—C3—O1	−177.00 (13)	C3—C4—C5—C6	0.2 (2)
C1—C2—C3—C4	2.1 (2)	C2—C1—C6—C5	−0.9 (2)
C8—O2—C4—C5	−93.85 (15)	C2—C1—C6—C10	175.88 (13)
C8—O2—C4—C3	87.29 (15)	O3—C5—C6—C1	178.16 (12)
O1—C3—C4—O2	−3.77 (19)	C4—C5—C6—C1	1.1 (2)
C2—C3—C4—O2	177.08 (13)	O3—C5—C6—C10	1.3 (2)
O1—C3—C4—C5	177.38 (12)	C4—C5—C6—C10	−175.69 (12)
C2—C3—C4—C5	−1.8 (2)	N1—C10—C6—C1	0.1 (2)
C9—O3—C5—C4	−87.17 (15)	N1—C10—C6—C5	176.74 (13)
C9—O3—C5—C6	95.76 (15)	C6—C10—N1—N1^i^	−178.28 (13)

Symmetry codes: (i) −*x*, −*y*+1, −*z*+1.

### Hydrogen-bond geometry (Å, °)

**Table:**

*D*—H···*A*	*D*—H	H···*A*	*D*···*A*	*D*—H···*A*
C7—H7B···O3^ii^	0.98	2.54	3.3620 (18)	142
C8—H8B···O1^iii^	0.98	2.62	3.3735 (19)	134
C8—H8B···O2^iii^	0.98	2.62	3.5711 (18)	164

Symmetry codes: (ii) *x*+1/2, −*y*+1/2, *z*+1/2; (iii) −*x*+3/2, *y*−1/2, −*z*+3/2.
